# Fetal-Perinatal Exposure to Bisphenol-A Affects Quality of Spermatozoa in Adulthood Mouse

**DOI:** 10.1155/2020/2750501

**Published:** 2020-03-20

**Authors:** Teresa Chioccarelli, Francesco Manfrevola, Marina Migliaccio, Lucia Altucci, Veronica Porreca, Silvia Fasano, Gilda Cobellis

**Affiliations:** ^1^Department of Experimental Medicine, Sez. Bottazzi, Università degli Studi della Campania “L. Vanvitelli”, Via Costantinopoli 16, 80138 Napoli, Italy; ^2^Department of Precision Medicine, Università degli Studi della Campania “L. Vanvitelli”, Via L. De Crecchio 7, 80138 Napoli, Italy

## Abstract

Bisphenol-A (BPA) is considered an endocrine disruptor with estrogenic activity. It is described as an environment-polluting industrial chemical whose adverse effects on the male reproductive system depend on the period of exposure (i.e., fetal, prepubertal, or adult life). We exposed male mice to BPA during the fetal-perinatal period (from 10 days post *coitum* to 31 days post *partum*) and investigated the impact of this early-life exposure on gamete health in adulthood animals at 78 days of age. Both in control and BPA-exposed mice, viability and motility of spermatozoa, as well as sperm motility acquisition and chromatin condensation of spermatozoa, have been evaluated. Results reveal harmful effect of BPA on viability and motility of sperm cells as well as on chromatin condensation status during epididymal maturation of spermatozoa. In particular, BPA exposure interferes with biochemical mechanism useful to stabilize sperm chromatin condensation, as it interferes with oxidation of thiol groups associated to chromatin.

## 1. Introduction

During spermiogenesis, round spermatids undertake an extensive morphological transformation useful to form elongated mature spermatids (SPT) whose chromatin is mainly packaged by protamines more than histones (about 5% in mouse). Spermiation promotes detachment of mature SPT, i.e., spermatozoa (SPZ) from the nursing Sertoli cells. Simultaneously, a rhythmic contraction of peritubular myoid cells surrounding seminiferous tubules propels SPZ until the *caput* region of epididymis [1]. Sperm maturation occurs during epididymal transit from *caput-*to-*cauda*, as SPZ acquire their potential motility and further remodel some cellular compartments and structures [2, 3]. In eutherian mammals, including mouse and human, inter-/intra-protamine disulphide bridges formation occurs during the epididymal transit. This event further condenses chromatin, closing it in a tighter structure [4].

The neuroendocrine axis hypothalamus-hypophysis-gonad finely regulates the production of mature and quality gametes. It is known that androgens and, more recently, estrogens too have a key role in such modulation [5, 6]. Any interference with the neuroendocrine axis interferes with gamete quality.

Endocrine disruptors are environmental contaminants with anthropogenic origin, able to interfere with the endocrine system. Bisphenol-A (BPA) is described as endocrine disruptors, used in the manufacture of plastics and other products, largely present in the environment, with estrogenic activity [7]. Noteworthy, BPA shows estrogenic, antiestrogenic, and antiandrogenic activities; it binds estrogen receptor (ER) alpha (ER*α*; with agonistic and antagonistic effects) or beta (ER*β*; with agonistic effects) and interferes with thyroid hormone signalling [8–10]. Furthermore BPA may also act via membrane estrogen receptor by producing effects that are similar in potency to those of estradiol (E2) [11].

Consistently, clinical, epidemiological, and experimental studies show that the origins of some male reproductive tract disorders (i.e., cryptorchidism, low sperm count and quality, and infertility) can be traced back to the intrauterine period and show that fetal-perinatal BPA exposure, during the critical developmental/differentiation stages, may derange mechanisms controlling fertility in both animals and humans [12]. In fact, BPA is able to cross the placenta, transferring to the fetus [13] and later to the neonate, via milk [14], significantly increasing the risk of developing chronic diseases in the adult life [15]. However, BPA may accumulate in the embryo/fetal compartment after repeat maternal exposure, most likely as fetus is not able to efficiently metabolize BPA [16]. In this way, BPA differentially affects the male reproductive tract. The extent of its effects depends on the period of exposure (i.e., fetal, prepubertal, or adult life) [17]. In mouse, prenatal exposure to BPA adversely affects spermatogenesis in adulthood animals, with a reduction in the number of seminiferous tubules at stage VIII that predisposes sperm count decrease [18]. Accordingly, gestational exposure to BPA is reported to decrease the proportion of elongated SPT [19] as well as the number of SPZ [20]. In addition, men with different degrees of fertility, classified as slightly, moderately, and severely infertile men, reveal a negative association between seminal BPA levels (but not BPA plasma levels) and sperm concentration or total sperm count and morphology [21]. More recently, it has been reported that BPA induces breaks in DNA strands and generate reactive oxygen species in SPZ [22–24]. Other studies report a detrimental, dose-dependent effect of BPA on selected fertility-related proteins in SPZ as well as on function and fertilization ability of sperm cells [25]. Although adverse effect of BPA on semen quality is consistent, there is insufficient evidence to draw conclusions about how BPA interferes with sperm health. Therefore, we performed an *in vivo* study to evaluate the possible effects of BPA exposure on SPZ. Specifically, we evaluated the impact of the fetal-perinatal exposure on gamete health in adulthood animals.

## 2. Materials and Methods

### 2.1. Experimental Design and BPA Exposure

The experimental design has been structured to avoid undesired environmental contamination of BPA. Accordingly, standard polypropylene cages (Tecniplast S.p.A., Varese, Italy), corncob bedding (Envigo srl, Udine, Italy), and glass bottles (Zooplus AG, Monaco di Baviera, Germany) were used [26].

Mice strain (CD1, Charles River), diet (Envigo srl, Udine, Italy), and BPA concentration (10 *μ*g/mL, Sigma-Aldrich, Milano, Italy), as well as route (drinking water), time (from 10 days *post coitum* (*dpc*) to 31 days *post partum* (*dpp*)) and period (foetal/perinatal) of exposure were chosen in agreement with effectiveness of the experimental plan reported by Miyawaki and coworkers [27]. In particular, we chose 10 *μ*g/mL as *in vivo* concentration according to “safe” dose of BPA for human by U.S. European Protection Agency.

Male mice were exposed to drinking water containing ethanol alone (0.2% as vehicle; *n* = 8, unexposed/control group; CTRL) or containing BPA (10 *μ*g/mL BPA dissolved in 0.2% ethanol; *n* = 12, exposed group), via pregnant/nursing mothers (from 10 *dpc* to 21 *dpp*) o via direct access to water (from 21-to-31 *dpp*).

After weaning, each male litter (*n* = 5 litters/CTRL group; *n* = 5 litters/BPA-exposed group) was housed in a single cage, and some physiological parameters (e.g., weight, food intake, etc.) were constantly monitored from 21-to-78 *dpp*, in both experimental groups [28]. The animals were sacrificed at 78 *dpp* and subjected to tissue collection. To note, before sacrifice, food was removed from the cage at 5:00 pm and animals were killed the day after, between 9:00 and 11:30 am, under ether anaesthesia by cervical dislocation. Epididymis were accurately removed, and the *caput* and *cauda* region were properly processed for SPZ collection from *caput* (*caput* SPZ) and *cauda* (*cauda* SPZ), separately.

Experiments were approved by the Italian Ministry of Education and the Italian Ministry of Health, with authorization *n*° 941/2016-PR issued on 10.10.2016.

Procedures involving animal care were carried out in accordance with National Research Council's publication *Guide for Care and Use of Laboratory Animals* (National Institutes of Health Guide).

### 2.2. Spermatozoa Collection

*Caput* and *cauda* SPZ (*n* = 5 for CTRL group; *n* = 5 for BPA group) were collected from the relative epididymal segment. In particular, *caput* and *cauda* of epididymis were separately immersed in phosphate buffer saline (PBS, pH 7.6) and cut into few pieces to let the SPZ flow out from the ducts. SPZ samples were then filtered and immediately used (*n* = 5 for the experimental group) to analyse the number of live, motile, and total cells. Aliquots of SPZ samples were fixed (*n* = 3 for experimental group) and later used to evaluate sperm chromatin parameters such as condensation and disulfide bound formation.

### 2.3. Analysis of Live and Motile Spermatozoa

We used CTRL (*n* = 4) and BPA-exposed (*n* = 4) mice to analyze the number of live and motile SPZ from *caput* and *cauda* of epididymis. The number of live and motile SPZ was evaluated under a light microscope (magnification 20X) using a haemocytometer (Burker Chamber). This procedure was validated using double-blind test. Live SPZ were evaluated using the viable dye tripan blue and plotted as percentage of live/total SPZ. Motile SPZ were count and plotted as percentage of motile/live SPZ.

### 2.4. Acridine Orange (AO) Staining Analysis

The fluorochrome AO intercalates into double strand DNA (native DNA) as a monomer and fluoresces green. Conversely, when it binds to single strand DNA (denatured or single strand DNA) as an aggregate, a red fluorescence is observed. Noteworthy, DNA is vulnerable to denaturation under acid conditions [29, 30]. This metachromatic shift from green (FL1-H) to red (FL3-H) has been used to measure chromatin quality indices of SPZ under acid conditions [31, 32].

Using cytofluorimetry analyses, we evaluated the percentage of SPZ with high DNA stainability (i.e., HDS) as well as thiol/disulphide status (i.e., TDS) in sperm samples collected from *caput* and *cauda* region of epididymis. Values were considered as spermatic indices of uncondensed chromatin (i.e., HDS, calculated as intensely green (FL1-H > 10^5^) fluorescing DNA/total fluorescing DNA (FL1-H > 10^3^ + FL3-H > 10^3^)) and thiol groups oxidation (i.e., TDS, calculated as red fluorescing [FL3-H > 10^3^]/green fluorescing (FL1-H > 10^3^) DNA), respectively [5, 29, 31, 32].

Aliquots of SPZ (1 × 10^6^/100 *μ*L) collected from *caput* or *cauda* epididymis were suspended in 1 ml of ice-cold PBS (pH 7.4) buffer and centrifuged at 600*g* for 5 minutes. The pellet was resuspended in ice-cold TNE (0.01 M Tris-HCl, 0.15 M NaCl and 1 mM EDTA, pH 7.4) buffer and again centrifuged at 600*g* for 5 minutes. The pellet was then resuspended in ice-cold TNE-10% glycerol buffer (200 *μ*L) and immediately fixed in ethanol (70% v/v) at 4°C for 24 h. Cytofluorimetry analysis was simultaneously carried out on *caput* and *cauda* SPZ from CTRL vs. BPA-exposed mice. The samples were treated for 30 seconds with 400 *μ*L of a solution of 0.1% Triton X-100, 0.15 M NaCl and 0.08 N HCl, pH 1.2. After 30 seconds 1.2 mL of staining buffer (6 *μ*g/mL AO, 37 mM citric acid, 126 mM Na_2_HPO_4_, 1 mM disodium EDTA, 0.15 M NaCl, pH 6.0) was admixed to the test tube and analyzed by flow cytometry. After excitation by a 488 nm wavelength light source, AO bound to a double-stranded DNA fluoresces green (515–530 nm) and AO bound to a single-stranded DNA fluoresces red (630 nm or greater). A minimum of 10,000 cells were analyzed by fluorescent activated cell sorting (FACSCalibur, BD BioScience, Milan, Italy).

### 2.5. Incubation of SPZ with BPA by *In Vitro* Experiment

Spermatozoa collected from *caput* epididymis of adult mice (*n* = 3) were treated with vehicle or with two different doses of BPA, 2 ng/mL and 20 ng/mL, corresponding to 0.01 *μ*M and 0.1 *μ*M, respectively. These doses are below the acceptable human daily exposure levels [33]. Each treatment lasted 15 min and was carried out at room temperature. Afterward, the number of total, live, and motile SPZ was evaluated as described above.

To preserve control quality and avoid any effect due to the vehicle, all experimental groups received ethanol (0.01%), being BPA dissolved in ethanol.

### 2.6. Statistical Analysis and Data Presentation

Student's *t*-test and Duncan's test (for multigroup comparison) were carried out to evaluate the significance of differences. Data are expressed as the mean ± S.E.M.

## 3. Results

### 3.1. BPA Effects on Viability of *Caput* Spermatozoa

Spermatozoa collected from *caput* epididymis of CTRL and BPA-exposed mice have been used to evaluate live SPZ. In particular, the number of live and total cells was analysed, and data were reported as percentage of live/total SPZ.

**Figure 1:**
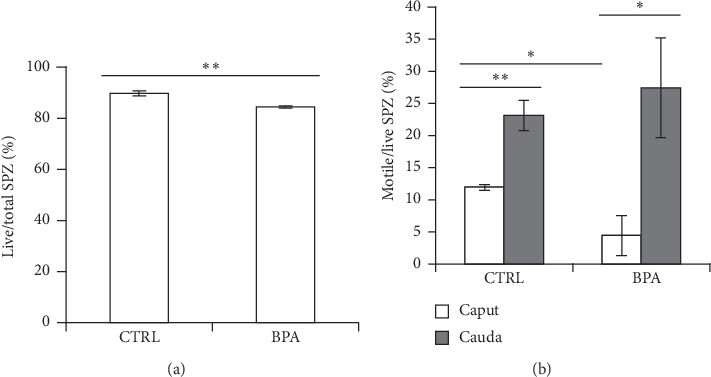
Viability (a) of spermatozoa (SPZ) collected from caput epididymis of mice exposed to vehicle (control, CTRL) or Bisphenol-A (BPA). (b) Motility of SPZ collected from caput and cauda epididymis of mice exposed to vehicle (CTRL) or BPA. Data are reported as percentage of motile/live SPZ ± S.E.M. ^*∗*^*p* < 0.05 and ^*∗∗*^*p* < 0.01.

The percentage of live SPZ (Figure 1(a)) from *caput* epididymis was significantly higher (*p* < 0.01) in the CTRL group as compared with BPA-exposed animals.

### 3.2. BPA Effects on Sperm Motility Acquisition

To study the interference of BPA on epididymal sperm maturation and, in particular, on sperm motility acquisition during the epididymal transit, from *caput*-to-*cauda*, we analyzed the percentage of motile SPZ from *cauda* and *caput* epididymis of CTRL and BPA-exposed mice (Figure 1(b)). In particular, the number of live, motile, and total cells was analysed, and data were reported as percentage of motile/live SPZ.

In CTRL and BPA-exposed mice, the percentage of motile SPZ significantly increased from *caput* to *cauda* (*p* < 0.01 or *p* < 0.05). The percentage of motile SPZ from *caput* epididymis was significantly higher (*p* < 0.05) in the CTRL group as compared with the BPA-exposed animals, while comparable percentage values of motile SPZ were observed in *cauda* epididymis of CTRL and BPA-exposed group.

### 3.3. BPA Effects on *Caput* SPZ by *In Vitro* Incubation

To investigate the harmful BPA effects on viability and motility of *caput* SPZ, we planned *in vitro* experiments and specifically incubated *caput* SPZ with vehicle (CTRL) or with two different doses of BPA (2 and 20 ng/mL). The number of live, motile, and total sperm cells was evaluated, and data were reported as the percentage of live/total SPZ or motile/live SPZ.

**Figure 2:**
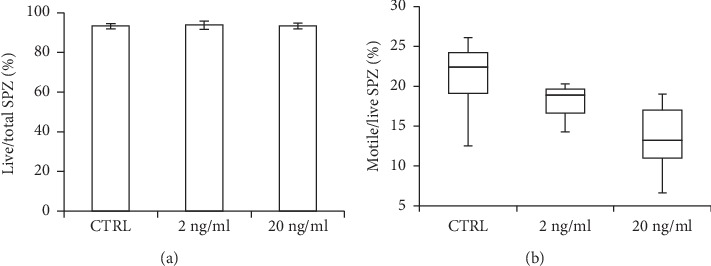
Viability (a) and motility (b) of spermatozoa (SPZ) collected from caput epididymis of adult mice incubated with vehicle (control, CTRL) or with two different doses of Bisphenol-A (BPA) (2 and 20 ng/ml). Data are reported as percentage of live/total SPZ (a) and motile/live SPZ (b) ±S.E.M. ^*∗*^*p* < 0.05.

Results show that viability of SPZ was unresponsive to the direct BPA exposure (Figure 2(a)). Indeed, both doses (2 and 20 ng/mL) were not able to decrease the number of live SPZ. Interestingly, the higher dose of BPA (20 ng/mL) significantly decreased the percentage of motile SPZ as compared to the CTRL group (*p* < 0.05), while no significant difference was observed at the lower doses (2 ng/mL) (Figure 2(b)).

### 3.4. Effects of BPA on Chromatin Condensation of *Caput* and *Cauda* Spermatozoa

Sperm samples from *caput* and *cauda* epididymis of CTRL and BPA-exposed mice have been stained with AO dye in acid conditions and analyzed by flow cytometry. HDS and TDS have been evaluated and used as spermatic indices of uncondensed chromatin and thiol-disulphide status, respectively.

**Figure 3:**
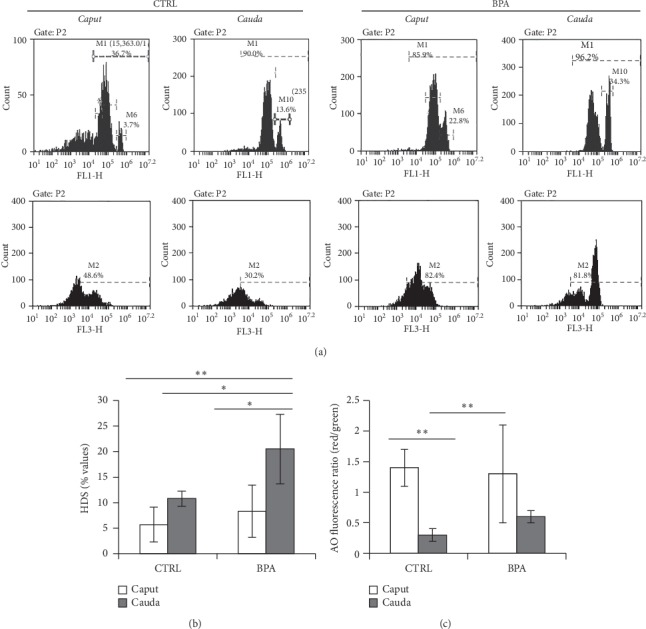
Flow cytometry analysis of caput and cauda spermatozoa (SPZ) from mice exposed to vehicle (control, CTRL) or Bisphenol-A (BPA). (a) Representative histograms of Acridine orange (AO) stained sperm in M1- and M2-gated areas. Intensely green (FL1-H > 10^5^), green (FL1-H > 10^3^), red (FL3-H > 10^3^), and total (green + red) fluorescencing DNA were used to analyze (b) high DNA stainability (HDS) and (c) thiol/disulphide status (TDS) values. Graphs were representative of three sperm samples relative to separate animals. Data were expressed as the mean ± S.E.M. ^*∗*^*p* < 0.05 and ^*∗∗*^*p* < 0.01.

Figure 3(a) shows histograms of green-stained (FL1-H) and red-stained (FL3-H) *caput* and *cauda* SPZ from CTRL and BPA-exposed mice, in the gated areas (M1 and M2, respctively), while the Figures 3(b) and 3(c) show the relative HDS and TDS values, respectively.

In CTRL mice, HDS values showed no significant difference, CTRL vs. BPA, while, in BPA-exposed mice significantly increased from *caput* to *cauda* epididymis (*p* < 0.05). However, in *cauda* epididymis, HDS values were higher in BPA-exposed mice than in the CTRL group (*p* < 0.05).

In CTRL mice, TDS values significantly decreased from *caput* to *cauda* epididymis (*p* < 0.05) while, in BPA-exposed mice, no significant difference was detected from the *caput* to *cauda* region.

## 4. Discussion

Male mice exposed to BPA during the fetal-perinatal period have been sacrificed at 78 *dpp*. Spermatozoa collected from *caput* and *cauda* epididymis have been morphologically and biochemically analyzed. The aim was to evaluate the impact of the fetal-perinatal exposure on gamete health in adulthood animals. Phenotypic parameters and chromatin features have been considered to qualitatively study the gamete as the result of events related to spermatogenesis and epididymal maturation. Phenotypic parameters of SPZ, such as viability and motility, have been evaluated by analysis of *caput* SPZ, while epididymal sperm maturation processes, such as motility acquisition and chromatin condensation, have been evaluated by analysis of *caput* and *cauda* SPZ.

Results demonstrate that BPA exposure decreased the number of live or motile SPZ in *caput* epididymis revealing adverse effects of BPA on viability and motility of sperm cells. Interestingly, BPA disturbed spermatogenesis as seminiferous tubules of BPA-exposed mice shown exfoliated germ cells in tubular lumen, particularly elongating SPT and SPZ (data not shown). However, despite in *caput* epididymis BPA decreased the number of motile SPZ, no effect was observed in the *cauda* region. Number of motile SPZ increased from *caput* to *cauda*, both in CTRL and BPA-exposed mice, with comparable values in *cauda*, CTRL vs. BPA, revealing that SPZ efficiently acquired their potential to move during the epididymal transit. This occurred independently by exposure, demonstrating that BPA did not interfere with sperm motility acquisition mechanism while it specifically counteracted sperm motility in *caput* epididymis.

To investigate the harmful effect of BPA on viability and motility of SPZ transiting in *caput* epididymis, using an *in vitro* incubation system, we directly exposed *caput* SPZ to vehicle or BPA (2 and 20 ng/ml) and analyzed the number of live or motile SPZ. No effect was observed on live sperm number, while a negative dose-dependent BPA-effect was noticeable on number of motile SPZ. This revealed that BPA was able to counteract sperm motility by direct action on SPZ, while it was not effective on viability. This observation, if translated in our *in vivo* model and, in particular, in *caput* epididymis of BPA-exposed mice, suggests that the higher number of dead SPZ was probably referable to harmful BPA-effect on germ cells during spermatogenesis. On the contrary, the lower number of motile SPZ was realistically ascribable to a local accumulation and action of BPA in the epididymal *caput* region. In agreement, the differential accumulation of endocrine disruptors, including BPA, has been reported in different animal tissues [34] including the visceral fat mass, which in our exposure experimental model preferably accumulates BPA [35]. Interestingly, the gestational BPA exposure adversely affects spermatogenesis in adulthood mice. Such exposure interferes with development of elongated SPT, as BPA likely acts as estrogenic chemical [19]. Indeed, estrogens lead to degeneration of elongated SPT in young rats *in vivo* exposed to high doses [36], while in mouse they modulate chromatin remodeling of SPT during spermiogenesis [37, 38]. In particular, 17*β*-Estradiol is reported to facilitate histone-protamine exchange in mouse SPT by promoting histone displacement [5]. Such event predisposes chromatin condensation extent of *caput* SPZ as well as predisposes *caput* SPZ to preserve chromatin condensation status during the epididymal transit by inter-/intra-protamine disulphide bridge formation [4].

With this in mind using a biochemical approach, based on AO fluorescent dye under acid conditions, we analyzed the percentage of SPZ with high DNA stainability (i.e., HDS) as well as thiol/disulphide status (i.e., TDS) in sperm samples collected from *caput* and *cauda* of epididymis of CTRL and BPA-exposed mice. Values were considered as spermatic indices of uncondensed chromatin (i.e., HDS) and thiol groups oxidation (i.e., disulphide bridges formation), respectively.

Results revealed scanty HDS values in *caput* epididymis, both in CTRL and BPA-exposed mice, with no significant difference, CTRL vs. BPA, demonstrating no effective BPA interference on spermatogenetic mechanisms that predispose chromatin condensation extent in *caput* SPZ. However, in *cauda* epididymis, the percentage of SPZ with uncondensed chromatin (i.e., HDS) was higher in BPA-exposed mice than in the CTRL group, suggestive harmful BPA effect on *cauda* SPZ. As expected, no significant differences was observed in the CTRL group when we analyzed HDS values in *caput* and *cauda* epididymis, confirming that SPZ preserve their chromatin condensation status during the epididymal transit. Conversely, in BPA-exposed mice, the HDS values increased from *caput*-to-*cauda*. This revealed higher susceptibility of sperm chromatin to swelling in *cauda* compared to *caput* epididymis. Noteworthy, during the epididymal transit of mouse sperm, inter-/intra-protamine thiol group oxidation strongly stabilizes sperm chromatin condensation status [4, 39, 40]. With this in mind, we hypothesized that BPA exposure interfered with epididymal sperm chromatin maturation by affecting disulphide bridge formation. In agreement, the thiol/disulphide ratio (i.e., TDS value) decreased in CTRL mice from *caput*-to-*cauda* SPZ, while in BPA-exposed mice, it was stably elevated in SPZ transiting from the *caput*-to-*cauda* region of epididymis, demonstrating that BPA-exposure counteracted the oxidation of the thiol groups associated to sperm chromatin.

In conclusion, our results show that mice exposed to BPA during the fetal-perinatal period produce poorly healthy gamete in adulthood. We show harmful effect of BPA on viability and motility of sperm cells as well as on sperm chromatin maturation extent. In particular, BPA interferes with biochemical mechanism useful to stabilize chromatin condensation of SPZ during the epididymal transit, by counteracting oxidation of thiol groups associated to chromatin.

## 5. Conclusions

The results of the current study support the hypothesis that endocrine-disrupting chemicals are important risk factors for declining male semen quality and suggest that environmental exposure to BPA may affect the main semen quality parameters. In the last few years, sperm quality parameters take on an important relevance in evaluation of gamete health. As an example, chromatin condensation and DNA damage in sperm cells [5, 40] are related each other, and DNA damage has been closely associated with poorer outcomes of numerous indicators of reproductive health, including lower fertilization, embryo quality, and implantation, and also with spontaneous abortion [41].

Of course, a clear understanding of BPA action mechanisms, including bioaccumulation, as well as of the presumed risks deriving from its exposure, is crucial to preserve male fertility. Moreover, given the complexity of BPA activity whose effects in animal models have been demonstrated at low and high doses [26], it is of critical public health importance to re-evaluate the current reference dose considered “safe” in humans [42].

## Data Availability

The data used to support the findings of this study are available from the corresponding author upon request.
